# Biomechanics of the lead straight punch of different level boxers

**DOI:** 10.3389/fphys.2022.1015154

**Published:** 2022-12-15

**Authors:** Yang Liu, Zhiqiang Zhu, Xiuxiu Chen, Chengyuan Deng, Xiujie Ma, Bin Zhao

**Affiliations:** ^1^ Sports Science Postdoctoral Research Station, Chengdu Sport University, Chengdu, China; ^2^ School of Physical Education, Nanchang Normal University, Nanchang, China; ^3^ Chinese Guoshu Academy, Chengdu Sport University, Chengdu, China; ^4^ School of Wushu, Chengdu Sport University, Chengdu, China; ^5^ Harbin Sport University, Harbin, China; ^6^ Shanghai Kongjiang Junior High School, Shanghai, China; ^7^ College of Physical Education and Health, East China Normal University, Shanghai, China

**Keywords:** different level, boxers, lead straight punch, technical effect, biomechanical characteristics, comparative study

## Abstract

We analyze and compare the differences in the biomechanical parameters between the lead straight punch and the index of force development of the lower extremities of boxers of different levels of ability. This can bridge the technical gap and provide insight and information for training strategies and athlete selection. To this end, a synchronized Vicon infrared 3D motion-capture system, two Kistler force platforms, and Kistler 8 target sensors were used for analysis. Sixteen boxers were recruited and sorted into an elite group (height 181.14 ± 3.01 cm, body mass 76.00 ± 10.028 kg) and a junior group (179.67 ± 5.84 cm, body mass 75.47 ± 12.19 kg), and their lead straight punch was then compared and analyzed. Three punch velocity indexes—peak velocity, contact velocity and Punching deceleration rate—six strength indexes—impulse, peak force, relative strength, peak time (frame), rate of force development (RFD), and movement time—and five exertion of both legs indexes— peak force, peak force/body mass, peak time, RFD index, and RFD/body mass index—were selected for analysis. Significant differences in the peak punch velocity and contact velocity were found between the two groups (7.162 ± 0.475 m•s^−1^vs. 6.317 ± 0.415 m•s^−1^, *Cohen’s d* = 1.89, *p* < 0.01, 5.557 ± 0.606 m•s^−1^ vs. 4.874 ± 0.385 m•s^−1^, *Cohen’s d* = 1.34, *p* < 0.05). Furthermore, significant differences were noted in the peak force [(1507.99 ± 411) N vs. (1035.45 ± 220) N, *Cohen’s d* = 1.43, *p* < 0.01], relative strength [(21.04 ± 5.88) N•kg^−1^ vs. (15.61 ± 2.53) N•kg^−1^, *Cohen’s d* = 1.19, *p* < 0.05], impulse [(88.61 ± 25.88) N•ms^−1^ and (60.53 ± 9.03) N•ms^−1^, *Cohen’s d* = 1.45, *p* < 0.05], and RFD [(88.61 ± 25.88) N•ms^−1^ and (60.53 ± 9.03) N•ms^−1^, *Cohen’s d* = 1.45, *p* < 0.05]. Among the four indexes of the lower extremities from two embedded Kistler force platforms, there were significant differences in the lead leg’s peak force/body mass [(19.68 ± 4.096) N•kg^−1^vs. (13.320 ± 2.223) N•kg^−1^, *t* = 3.902, *Cohen’s d* = 1.92, *p* < 0.01], RFD index [(16.90 ± 3.269) N•ms^−1^vs. (10.28 ± 4.313) N•ms^−1^, *Cohen’s d* = 1.72, *p* < 0.01], and RFD/body mass index [(23.47 ± 4.09%) N•ms^−1^Kg^−1^ vs. (15.38 ± 5.65%) N•ms^−1^Kg^−1^, *Cohen’s d* = 1.64, *p* < 0.01]. There were no significant differences in the four indexes on the rear leg between the two groups (*p >* 0.05). Based on the disparity in the effect of the lead straight punch and the biomechanical parameters of both lower extremities, the boxers must attach importance to sequential acceleration-braking training to improve the terminal velocity of the hand, and thus improve the contact velocity. Furthermore, it is advised that coaches and practitioners carefully consider increasing start-up strength training of the lead leg and attempt to improve the peak velocity of the lead straight punch. In addition, these biomechanical parameters can be used as criteria for the selection of boxers.

## Introduction

The straight punch is a basic boxing technique and is the most common employed in Olympic boxing (Davis et al., 2018). It is split into two categories: the lead straight punch and the rear straight punch (Davis et al., 2015), of which the former generally delivers a knock-out blow to the opponent. Compared to the hook and upper-cut, the straight punch has the advantage of being faster and more effective (Piorkowski et al., 2011). As reported by previous studies, the lead straight punch, with changing rules of boxing, has become increasingly important in boxing matches and is used most frequently for both attack and defense. Boxers with superior straight lead techniques can throw punches faster and more powerfully than those who throw rear straight punches—the former is more efficient for scoring points by effectively hitting an opponent. Chinese boxer Gu Hong is well versed in the lead straight punch and can throw punches rapidly to disrupt her opponent’s attack, allowing her to stay ahead in a game. In the 2020 Olympics in Tokyo, Japan, she won a silver medal in the women’s 69 kg category. In combat sports, victory or defeat depends on a number of factors, such as the fighter’s endurance, technique, tactics, psychological characteristics, and the proficiency of the referee (Falco et al., 2009). Among these, punch velocity and strength are key in evaluating a boxer (Smith et al., 2000; Walilko et al., 2005). For training theory to make specific techniques economical and effective (Pierce et al., 2007), we must first ascertain the influence factors on the disparity between elite and junior groups to effectively revise training strategies. Few studies, however, have examined this disparity in terms of striking effectiveness and the biomechanical parameters of both the lower extremities’ force indexes of boxers of different levels. We therefore aim to reveal the biomechanical differences in the punch velocity, punch strength, and start-up strength of both the lower extremities of two groups of boxers of different skill levels, in order to provide advice for junior boxers on scientific training and how to bridge the technical gap.

## Materials and methods

### Subjects

A total of 16 males were selected from the boxing team of the competitive sports school affiliated with the Shanghai University of Sports. Based on the level of athletic competition, we define athletes above national level as elite group players and Level II as junior athletes. The elite and junior groups’ mean age was 24.33 ± 1.75 and 17.11 ± 1.26 years old, respectively. All 16 subjects were numbered S1–S16, and various data were recorded. There was no statistically significant difference in height and body weight between the elite group [181.14 ± 3.01 cm, 76.00 ± 10.028 kg] and junior group [179.67 ± 5.84 cm, 75.47 ± 12.19 kg] (*p* > 0.05). Furthermore, the boxers involved were strictly screened, with those failing to meet such conditions excluded for the sake of group homogeneity (Table 1).

**TABLE 1:** Basic information on the boxers.

	Elite group	Junior group	*p*
Height (cm)	181.14 ± 3.01	179.67 ± 5.84	0.602
Body weight (kg)	76.00 ± 10.02	75.47 ± 12.19	0.535

### Protocol

The study design was to use two groups of boxers with different levels of technical biomechanical ability, using biological parameters to compare the technical characteristics and differences between the elite and junior groups in order to guide the training and selection of athletes. Prior to being conducted, this experiment was reviewed by professors and experts from the School of Sports Science, Shanghai University of Sport. The experiment was carried out in strict accordance with the Helsinki Declaration and approved by the Ethics Committee of the Shanghai University of Sport (approval number: 102772019RT033). The experiment’s location was the experimental hall of the School of Sports Science in March 2019; the temperature there was controlled at 25°C. Before entering the test, participants were required to sign an informed agreement, including no fatigue, injury, drinking, and physical illness, and were familiarized with the experimental protocol. Prior to physical fitness testing, a standardized warm-up protocol (i.e., 15 min of dynamic stretching, and running) was performed. The hand (R or L) point was pasted on the middle position of the back of the fist with double-sided 3M adhesive and was used to was used to mark the coordinate position to track the fist and calculate its velocity. Uniform boxing gloves were worn. During the test, to properly adjust the distance of the force target, subjects stood on two embedded Kistler force platforms on the left and right in the preparation position and struck with full force using the dominant limb (arm). Boxers were tested with three effective straight punches, taking the performance of the best straight punch.

### Instruments and equipment

**FIGURE 1:**
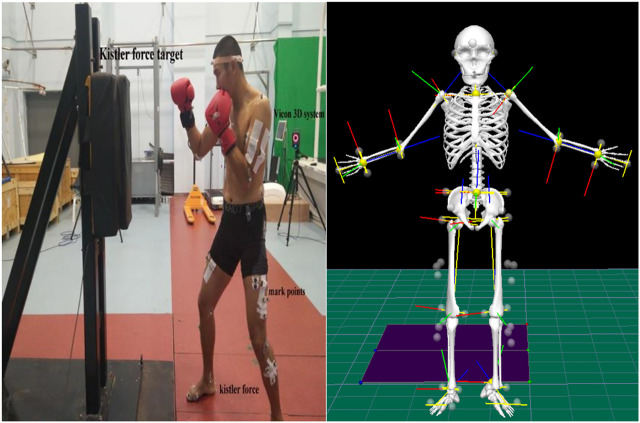
Boxer preparation posture with Kistler’s force target fixed on tripod and two force gauges on both legs.

The study used one Kistler force target (Switzerland, 1000 Hz) fixed on a tripod to record the strength of striking and two embedded Kistler force platforms (Switzerland, 9287B, 90×60 cm, built-in signal amplifier, 1000 Hz) to record the change in the strength of the subjects’ legs. The Vicon nexus with 16 cameras (2.6.1, Vicon motion analysis Inc. 200 Hz) and reflective marking balls were used to mark individual joints to modeling. The Vicon device was used to record the kinematic data of the subjects, calculate the displacement, and derive the first-order derivative to obtain velocity. The digital signals collected by the Vicon motion capture system and the Kistler 3D-force measuring platform were converted into synchronized analogue signals using a synchronization device that collects the pulse signals of three instruments at the same time (frame). These instruments have been used in most of the literature and have proven to have good reliability and validity. In a pilot study, we repeatedly checked the quality of the devices in the lab (Figure 1).

### Index selection and data processing

#### Index selection

**FIGURE 2:**
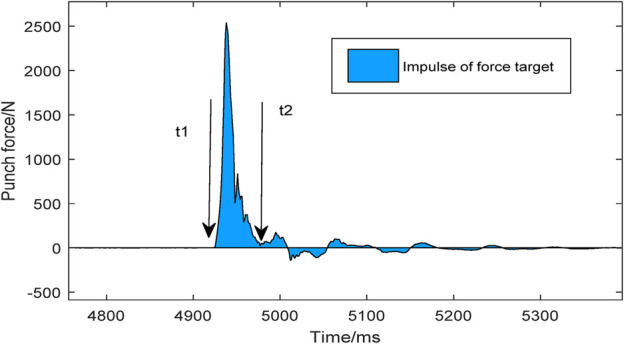
The impulse of punching.

The comparison Kistler target index mainly includes the force target index, punch velocity index, and the punch force index (Figure 2).

① Kistler Force Plate for the Punching Index

The impact impulse is defined as the area under the curve of the force and time-formed when the fist contacts and leaves the Kistler target. Mathematically, this is the integral of force F (t) with respect to time (Figure 2: t_1_-t_2_).

$$I=\int_{t1}^{t2}F\left(t\right)dt$$

The peak force is defined as the force-value curve interaction time action to the peak when the fist contacts the force-measuring target. The impact force rate is defined as the linear data peak force of the force-measuring target divided by the time to reach peak force.

② Ground Reaction Force for Both Legs Index

Three-dimensional ground reaction force data were derived from the embedded force platform through the Kistler force plate. The subjects stood with both legs on the embedded force platforms to record the changes in the start-up strength of both legs. The rate of force development (RFD) is the peak force/peak time (F_max_/T_max_) (Suchomel et al., 2014;Aeles et al., 2022). RFD also defines the lower-limb index selections of the start-up strength—RFD/body mass, peak force, and time of peak force.

③ Punching Velocity Index

The peak punch velocity, contact velocity, punch velocity decay rate, and other indexes were selected. The peak punch velocity is the peak point on the velocity time series curve after the mark point displacement of the marked punch and is time-derivative. The calculation of contact velocity can be modeled by V3D software, and the previous frame is synchronized when the fist hits the Kistler force target. The velocity is obtained by taking the displacement derivative of the mark point (Hand), and the acceleration can be obtained by taking further derivatives of the velocity. The index calculation method of the velocity deceleration rate is as follows:

$$Deceleration\;rate=\frac{Peak\;velocity-Contact\;velocity}{Peak\;velocity}$$

### Data processing and V3D modeling

This study used Vicon Nexus software to calculate missing mark points; the data after dot filling was collected in CMO format that was imported into V3D software for human modeling (Figure 1). Kinematics and dynamics data were then analyzed. The “start event” and “finish event” tags were defined to gain export kinematics and GRF data in ASCII format for further analysis in Microsoft Excel. The punching force data of the Kistler force target must only analyze the *Z*-axis data (vertical data) using Bioware software. The lower limbs used three axes of the direction of the combined force $F=\sqrt{x^{2}+y^{2}+z^{2}}$ .

### Statistical analysis

The sample size for significant observations were calculated in G ∗Power. Relevant data can be imported into SPSS24.0 for analysis, and all data are subjected to the single-sample Kolmogorov–Smirnov (K-S) test to verify whether they obey the normal distribution. The data for all compared indicators were tested as obeying the normal distribution. Descriptive statistics are represented as *M ± SD*. The independent sample *t-test* was used to compare the differences in various indexes of the lead straight punch techniques of athletes of different levels (for parametric tests). The significance level was set at *p* < 0.05. The standardized effect size (*Cohen’s d*) was used to interpret the magnitude of the difference between the lead punch and rear punch data. A common interpretation of effect sizes is small (*d* = 0.2), medium (*d* = 0.5), and large (*d* = 0.8), based on the work.

## Results

### Analysis of force Kistler target

**FIGURE 3:**
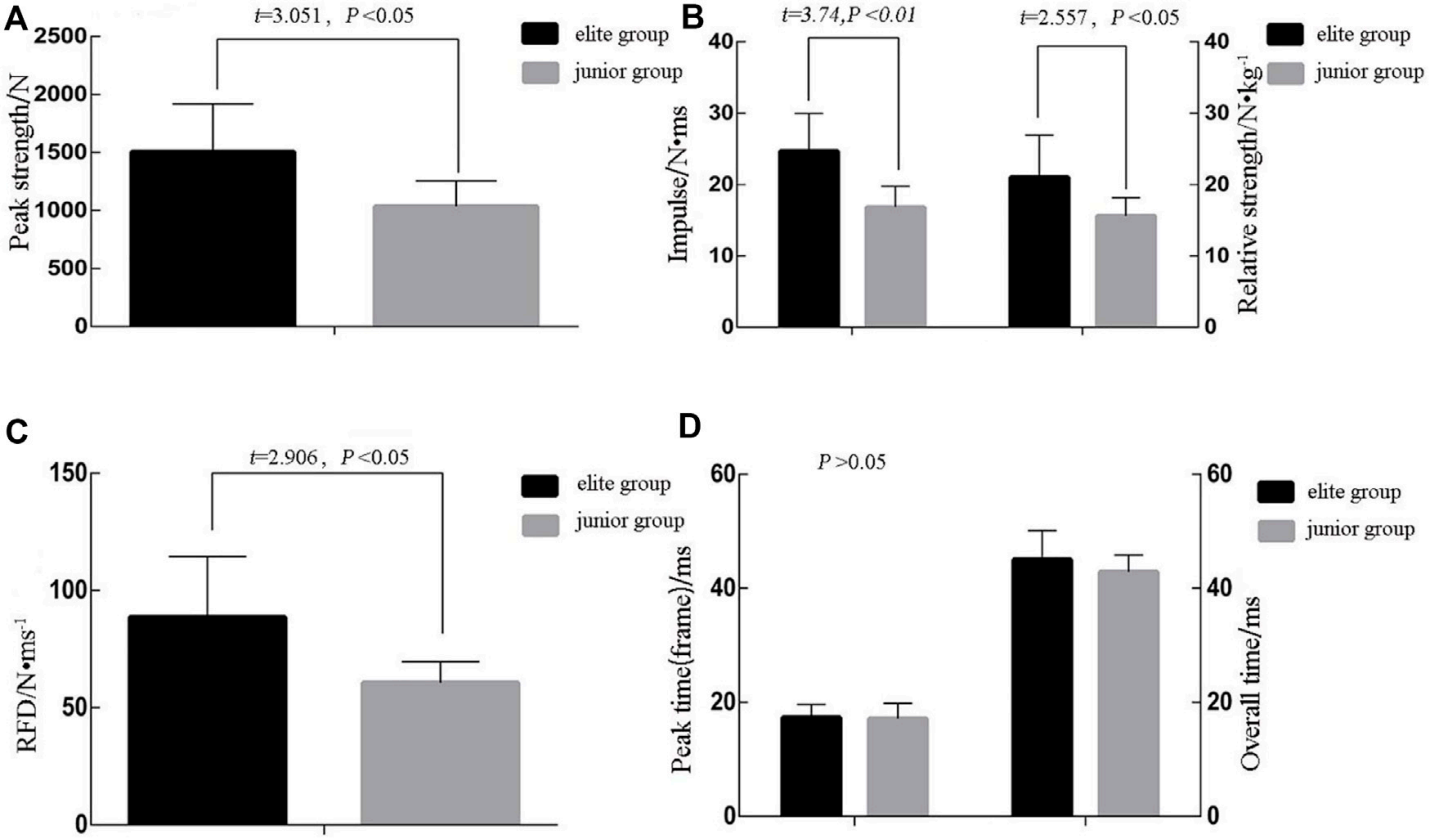
Statistical analysis of strength measuring target indexes.

Six secondary indexes—the impulse, peak force, relative strength, peak time (frame), RFD, and movement time—were selected from the Kistler target for data presentation and comparative analysis. Four indexes showed statistically significant differences between the elite and junior group. The elite group, compared to the junior group, had higher punch impulses [(24.70 ± 5.26) N•ms vs. (16.82 ± 2.92) N•ms, *t* = 3.74, *Cohen’s d* = 1.85, *p* < 0.01], peak force [(1507.99 ± 411) N vs. (1035.45 ± 220) N, *t* = 3.051, *Cohen’s d* = 1.43, *p* < 0.01], relative strength [(21.04 ± 5.88) N•kg^−1^ vs. (15.61 ± 2.53) N•kg^−1^, *t* = 2.557, *Cohen’s d* = 1.19, *p* < 0.05], and RFD [(88.61 ± 25.88) N•ms^−1^ and (60.53 ± 9.03) N•ms^−1^, *Cohen’s d* = 1.45, *t* = 2.906, *p* < 0.05]. The other two indexes were not significantly different between the elite and junior group—overall time [(45.00 ± 5.148) ms vs. (42.88 ± 3.357) ms, *t* = 0.172, *p* > 0.05] and peak time (frame) [(17.33 ± 2.291) ms vs. (17.13 ± 2.696) ms, *t* = 0.993, *p* > 0.05] (Figure 3).

### Analysis of punching velocity index

**FIGURE 4:**
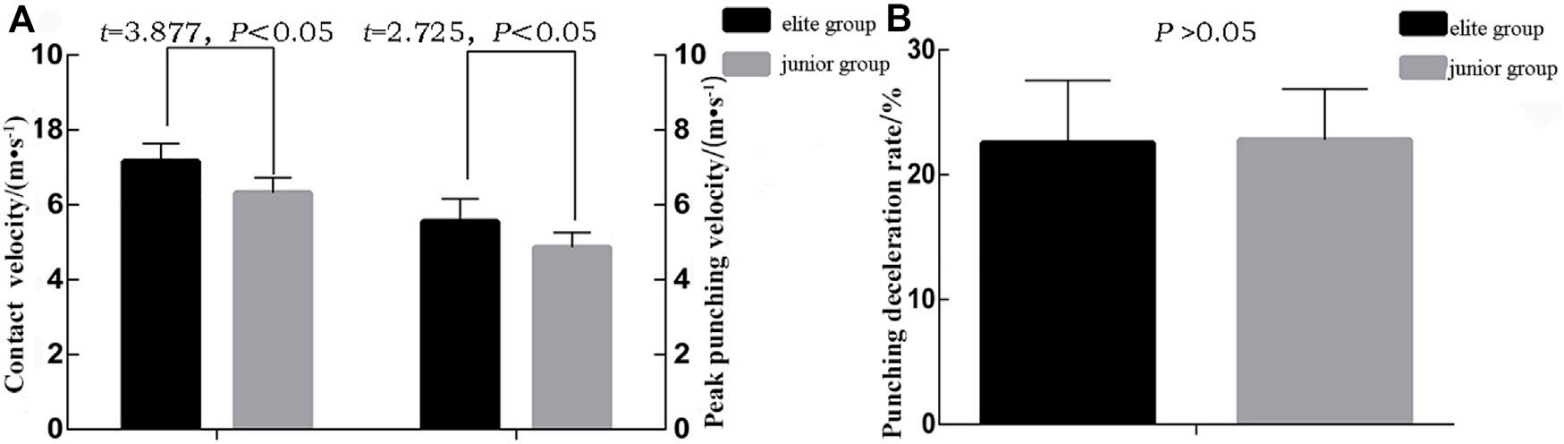
Statistical analysis of peak velocity, contact velocity, and punching deceleration rate indexes.

The boxers in the elite and junior groups had significant differences in peak punching velocity [7.162 ± 0.475 vs. 6.317 ± 0.415 m•s^−1^, *t* = 3.877, *Cohen’s d* = 1.89, *p <* 0.01] and contact velocity [5.557 ± 0.606 vs. 4.874 ± 0.385 m•s^−1^, *t* = 2.725, *Cohen’s d* = 1.34, *p* < 0.05], with the elite group achieving higher values than the junior group. The punching deceleration rate was not significantly different between the two groups [22.53 ± 5.03 vs. 22.80 ± 4.10, *t* = 0.295, *p* > 0.05] (Figure 4).

### Analysis of Kistler instrument index

**FIGURE 5:**
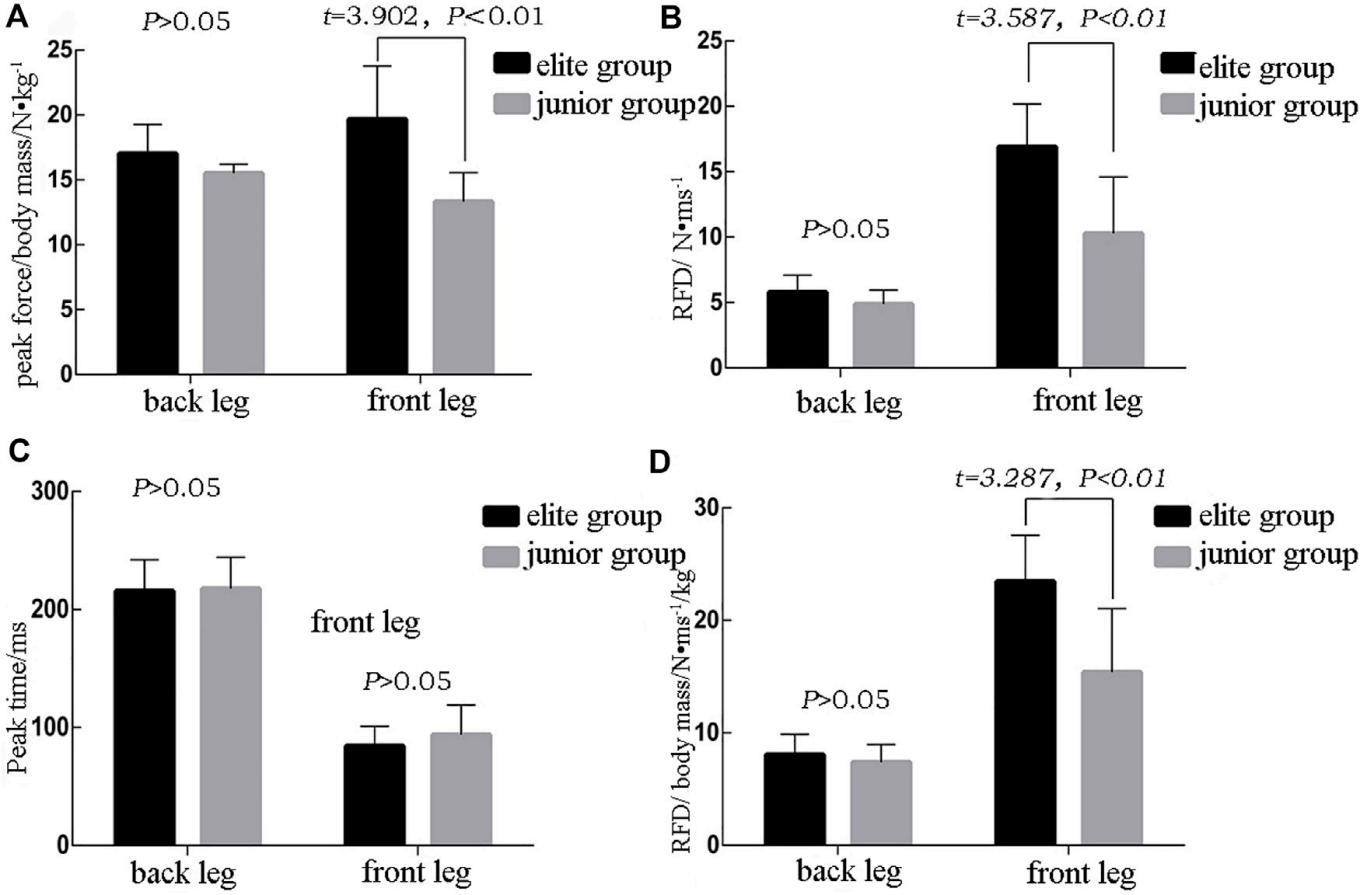
Analysis of force index results of lower limbs.

Five indexes were selected from the Kistler data of lower extremities strength for analysis: the peak force, peak force/body mass, peak time, RFD index, and RFD/body mass index. Between the elite and junior groups, there were five indexes of the lead leg start-up strength that were statistically significant, of which four were higher in the elite than in the junior group: the peak force/body mass [(19.68 ± 4.096) N•kg^−1^vs. (13.320 ± 2.223) N•kg^−1^, *t* = 3.902, *Cohen’s d* = 1.92, *p* < 0.01], RFD index [(16.90 ± 3.269) N•ms^−1^vs. (10.28 ± 4.313) N•ms^−1^, *t* = 3.587, *Cohen’s d* = 1.72, *p* < 0.01], and rapid force index/body mass [(23.47 ± 4.09%) N•ms^−1^Kg^−1^ vs. (15.38 ± 5.65%) N• ms^−1^Kg^−1^, *t* = 3.287, *Cohen’s d* = 1.64, *p* < 0.01]. However, there was no significant difference in the peak time between the elite group and the junior group [(84.44 ± 16.667) ms vs. (93.75 ± 25.306) ms, *p* > 0.05] (Figure 5).

Three of the five force indexes for the rear leg showed no significant difference between the two groups—peak force/body mass [(17.04 ± 2.24) N•kg^−1^ vs. (15.50 ± 0.71) N•kg^−1^, *t* = 1.847, *p* > 0.05], RFD index [(5.804 ± 1.292) N•ms^−1^ vs. (4.878 ± 1.093) N•ms^−1^, *p* > 0.05], and RFD/body mass index [(8.07 ± 1.80%) N• ms^−1^kg^−1^, vs. (7.39 ± 1.57%) N•ms^−1^kg^−1^, *p* > 0.05). The peak time of the leg was (215.56 ± 26.51) ms and (217.50 ± 43.34) ms, *p* > 0.05, threshold time [(437.78 ± 64.95) ms and (447.50 ± 52.30) ms, *p* > 0.05] (Figure 5). Overall, there was a significant difference in the positive force index of front legs between the elite and junior group.

## Discussion

### Punching velocity indexes of boxers at different levels

This study measured boxing velocity using the Vicon Nexus (Version 2.6.1, sampling frequency 200 Hz) motion capture system with 16 cameras and reflective marker balls to mark individual joints for modeling purposes. The Vicon infrared motion capture system records the displacement characteristics of the mark point. The three indexes of punch velocity were the peak velocity, contact velocity, and deceleration rate. Statistical tests revealed significant differences (*p* < 0.05) between the two groups in the indexes of peak and contact velocities, with the elite group significantly higher than the junior group. In combat sport, the rear hand (further from the target) usually has more punching strength, while the lead hand (closer to the target) can achieve the maximum velocity (Dyson et al., 2008). The lead straight punch is faster and more sudden owing to its short distance from the opponent and smaller angle of trunk rotation. Although the lead punch is not as powerful as the rear-hand punch, its continuity, accuracy, and effectiveness are not inferior.

Faster punches can do more “damage” to the opponent (Mcgill et al., 2010; Cheraghi et al., 2014). The results of this study showed that the elite boxers had higher peak punching and contact velocities and lower deceleration rate. The contact punch velocity of the elite group was significantly higher than the junior group [(5.557 ± 0.606 m•s) vs. (4.874 ± 0.385 m•s), *p* > 0.05]. Therefore, the junior group must focus on improving their contact punch velocity, which refers to the velocity of the “fist” at the frame before it contacts the target in space. The contact velocity determines the effect of a punch, and the peak velocity directly affects the contact velocity indexes. Unlike professional boxers, amateurs tend to strike the scoring area rather than striking hard blows, which is the distal segment maximization principle in biomechanical terms. To develop maximum velocity at the distal part of the kinetic chain, the distal segment maximization principle is consistent with limb element interactions, as in sports that involve throwing and kicking such as baseball, tennis, golf, and rugby.

The straight punch technique is an open kinetic chain movement, in which the proximal part of the limb is fixed and the distal part is active, with the limb segment (torso, arms, forearms, hands) accelerating and braking in sequence from the proximal to the distal segment, thus increasing the velocity and momentum of the distal segment (Cabral et al., 2010). According to the principle of maximizing the velocity of the distal segments, boxers of the junior group must focus on acceleration and braking training involving shoulder, elbow, and wrist joints sequentially accelerating and braking. This can increase the distal segment velocity of the hand, thus increasing the contact velocity and the momentum of the distal element.

In conclusion, appropriately increasing peak velocity can improve contact velocity, having a significant impact on the punching effectiveness. Boxers of the junior group who focus on acceleration and braking training can increase the distal segment velocity of the hand and the momentum of the distal segment, which has substantial implications for training instruction.

A Kistler target (Swiss, 1000 Hz) fixed to a tripod was used to record the force-time curve of the punch contacting and leaving the target. Both domestic and international literature report on the use of Kistler force target or accelerometers to measure the effects of boxing punches; however, there are several discrepancies in the measurement results (Buśko et al., 2016) (Pilewska et al., 2017; Walilko et al., 2005; Smith et al., 2000; Lenetsky et al., 2018; Lenetsky et al., 2016; Buśko et al., 2014; Smith et al., 2000). The Kistler platform boasts high reliability and validity. Boxing is a highly demanding physical-quality combat in which boxers rely on the interplay of qualities such as strength, coordination, velocity, and endurance to confront their opponents and avoid their attacks. During the rounds, the boxer’s goal is to knock out the opponent by punching the optimal target area to win the fight. A knockout is the consistent goal for boxers in a fight (Loturco et al., 2016). Boxers must thus increase the impact of their punches. This study examined the peak force, relative strength (Bolander et al., 2009; Vaslin, 2005), and RFD of the lead straight punches of boxers of the two different-level groups. The peak force that represents the peak value in the power curve of the elite group is significantly higher than that of the junior group [(1507.99 ± 411) N vs. (1035.45 ± 220) N, *t* = 3.051, *p* < 0.01].

Relative punch strength (N/kg) (Dunn et al., 2019) is meaningful in evaluating the striking effectiveness of different-level boxers (Halperin et al., 2016; Buśko et al., 2014), with the elite group significantly higher than the junior group [(21.04 ± 5.88) N•kg^−1^ vs. (15.61 ± 2.53) N•kg^−1^, *t* = 2.557, *p* < 0.05). Compared to the junior group, the elite group delivered more impulse to the Kistler target (24.70 ± 5.26) N•ms vs. 16.82 ± 2.92 N•ms), which could be attributed to high-level boxers producing more “effective mass”. According to the law of momentum, when the punching velocity is constant, the greater running mass (m) of the impacting object leads to a greater momentum (mv) delivered to the target. The “effective mass” of a punch affects its impact (*r* ^2^ = 0.45). As reported in several studies, there are only small linear relationships between different body mass categories and effective mass (*r* = 0.432, *p* = 0.074) (Walilko et al., 2005). Pain and Challis (2002) found that muscle activation during punching presents a “secondary pulse” phenomenon, and that EMG shows a “double peak” in muscle activation—said to increase limb “stiffness” and result in greater effective mass (Mcgill et al., 2010). In addition, Pain and Challis (2002) reported that arm positioning and the “second pulse” reduced energy loss during striking and enhanced effective hitting mass. It has been further reported by Walilko et al. (2005) that flexing the wrist prior to fist collision reduces the transfer of effective mass. The effective mass is the mass of the punching body involved in the transfer of momentum during the punch, taking into account the influence of the human body during the punch (Pierce et al., 2007; Lenetsky, et al., 2015).

How can the “effective mass” of the punch be improved for junior boxers (Walilko et al., 2005)? This study suggests that the “effective mass” of the punch depends on the mass of the striking upper limb (i.e., the cross-sectional area of the muscles) and on the ability to increase the effective mass transfer of the striking body when punching (i.e., throwing a punch with full power). When muscles contract, strength and stiffness are increased and the strike strength is maximized (Mcgill et al., 2010; Pain and Challis, 2002). The striking mass of a punch at the highest velocity of the blow is, at most, the mass of one arm (Neto and Magini, 2007). The rigid connecting structure formed by the upper arm, torso, and lower limb at the moment of strike can increase the mass and strength of the strike (Lenetsky et al., 2013).

In conclusion, to design special technical training for the lead straight punch, junior boxers should pay more attention to the connection structure of stiffness of the upper extremities, torso, and lower extremities at the moment of a punch to increase the instantaneous “stiffness” of the limb segment and the mass of effective striking, and thus improve the striking effect.

### Lower limbs indexes of boxers at different levels

Two embedded Kistler force platforms (Swiss, 9287B, length × width: 90 × 60 cm, built-in signal amplifier, 1000 Hz) were used to record the strength changes in the legs of boxers during the delivery of a lead straight punch. Some studies typically use two Kistler force platforms (Stanley et al., 2018) to measure the changes in the ground reaction force (GRF) of both legs (Lenetsky et al., 2020). There are numerous studies on the relationship between lower extremity movement index and upper extremity punching performance. The lower limb RFD index (peak force/force time) is an index of RFD. Zhang (2008) studied the correlation coefficient between the instantaneous RFD and off-ground velocity of top high jumpers, which reaches up to 0.965. In a boxing study, Su Yan Ju (2013) used two Kistler force platforms to investigate the correlation between the rear leg force index and punching velocity, achieved a coefficient reaching up to 0.882. The peak force/body mass of both legs and RFD/body mass were positively correlated to the velocity range of the punch. In some relevant biomechanics literature on the lead straight punch, delivering a lead straight punch requires start-up strength from the lead leg, which is dominant in the process of both lower limbs’ force development (Lenetsky et al., 2020). In actions such as striking, the body being struck, the striking body, and the ground form a complex mechanical system, where the lower extremities are in direct contact with the ground, playing an important role in the force development process.

There was no significant difference between the two groups in terms of all indexes of rear leg force development (*p* > 0.05), while the two groups significantly differed in lead leg power force development (*p* < 0.05). The difference between the two groups in terms of the biomechanical indexes of rear leg force development was not significant, but it cannot be said that rear leg force development is not important.

In the lower extremities strength indexes of two groups, the elite group exhibited a significantly higher lead leg peak force/body mass, lead leg strength index, and lead leg strength index/body mass than the junior group (*p* < 0.05). Therefore, junior boxers must develop skills of start-up strength in the lead leg appropriate to bridging the gap in lead straight punching effectiveness.

In conclusion, there were significant differences in the biomechanical parameters of the lead leg force development indexes between the two groups. When developing boxers’ lead straight punch technique, the ability to rapidly coordinate the force of lower lead leg is of great significance in bridging the technical gap.

## Conclusion

In the training of junior boxing athletes, attention must be paid to the velocity acceleration and braking training in each segment of the lead straight punch to increase the distal segment velocity of the hands as well as the contact velocity of the punch. It is recommended that coaches and practitioners carefully consider increasing the start-up strength training of the lead leg and attempt to improve the peak velocity of the lead straight punch. To this end, training such as unilateral SSC training and plyometric resistance training is necessary.

## Data Availability

The original contributions presented in the study are included in the article/Supplementary Material; further inquiries can be directed to the corresponding author.
